# Renal dysplasia characterized by prominent cartilaginous metaplasia lesions in VACTERL association: A case report

**DOI:** 10.1097/MD.0000000000006499

**Published:** 2017-04-14

**Authors:** Takeo Nakaya, Taiju Hyuga, Yukichi Tanaka, Shina Kawai, Hideo Nakai, Toshiro Niki, Akira Tanaka

**Affiliations:** a Department of Pathology, Jichi Medical University; b Department of Pediatric Urology, Children's Medical Center Tochigi and Jichi Medical University, Shimotsuke, Tochigi; c Department of Pathology, Kanagawa Children's Medical Center, Yokohama, Kanagawa, Japan.

**Keywords:** prominent cartilaginous lesions, renal dysplasia, VACTERL association

## Abstract

**Background:**

Renal dysplasia is the most important cause of end-stage renal disease in children. The histopathological characteristic of dysplasia is primitive tubules with fibromuscular disorganization. Renal dysplasia often includes metaplastic cartilage. Metaplastic cartilage in renal dysplasia has been explained as occurring secondary to vesicoureteral reflux (VUR). Additionally, renal dysplasia is observed in renal dysplasia-associated syndromes, which are combinations of multiple developmental malformations and include VACTERL association.

**Case presentation:**

We observed the following multiple developmental malformations in a 108-day-old male infant during a nephrectomy: a nonfunctioning right kidney with VUR, hemidiaphragmatic eventration, a ventricular septal defect (VSD) with tetralogy of Fallot in the heart, cryptorchidism, and hyperdactylia. These developmental anomalies satisfied the diagnostic criteria for VACTERL association. A surgical specimen of the right nonfunctioning kidney revealed prominent cartilaginous metaplasia in the renal dysplasia with VUR. The densities of the ectopic cartilaginous lesions in this nonfunctioning kidney were extraordinarily high compared with other renal dysplasia cases. Giemsa banding of his genome produced normal results. The patient has not undergone further detailed genomic investigation.

**Conclusion:**

This case might be a novel type of VACTERL association, that is, renal dysplasia combined with prominent cartilaginous metaplasia, tetralogy of Fallot and VSD of the heart, hemidiaphragmatic eventration, and hyperdactylia.

## Abstract 2

Supplemental Digital Content is available in the text

## 1 Introduction

Renal dysplasia with or without vesicoureteral reflux (VUR) is the most important cause of end-stage renal disease in children.^[1]^ Renal dysplasia is defined by abnormal metanephric differentiation.

The prevalence of renal dysplasia in infants is not rare. An autopsy study revealed a prevalence of renal dysplasia of 4% in infants and fetuses.^[2]^ An ultrasound study revealed a prevalence of renal dysplasia in infants of 0.1%.^[3]^ Moreover, renal dysplasia occurs with a prevalence of 3.7%, as revealed in a study of benign nonfunctioning kidneys that were removed by nephrectomy.^[4]^

The histopathological characteristic of renal dysplasia is primitive ducts with fibromuscular disorganization. Renal dysplasia is often accompanied by metaplastic cartilages, bones, and proliferating nerves.^[2]^

Metaplastic cartilage in renal dysplasia has been explained as occurring secondary to VUR.^[1]^

We observed renal dysplasia with VUR in a male infant that was characterized by prominent cartilaginous foci. The densities of the ectopic cartilaginous lesions in the nonfunctioning kidney of this case were extraordinarily high compared with those in typical renal dysplasia cases. Additionally, this case exhibited multiple accompanying developmental malformations that satisfied the diagnostic criteria for VACTERL association (at least three of the following congenital anomalies: vertebral defects [V], anorectal malformations [A], cardiac defects [C], tracheoesophageal fistula with or without esophageal atresia [TE], renal malformations [R], and limb defects [L]).^[5,6]^ These findings suggest that the prominent cartilaginous lesions might not have been exclusively due to VUR.

Here, we illustrate the case and discuss the histopathological features and origin of prominent cartilaginous foci in renal dysplasia with VUR against the background of VACTERL association.

## 2 Case report

### 2.1 Clinical history

The male infant patient was born at 39 weeks by Caesarian section. At 19 weeks into the pregnancy, ultrasonography revealed pyelectasis of the right kidney, a ventricular septal defect (VSD) of the heart, and a single umbilical artery.

At birth, the patient exhibited pale skin, nasal alar breathing, and mild groaning. His Apgar score was 8-8-8. His birth weight was 2284 g.

He suffered from multiple developmental malformations as follows: right hemidiaphragmatic eventration, tetralogy of Fallot, VSD in the heart, and a patent foramen ovale. He also had pulmonary hypertension.

**Figure 1:**
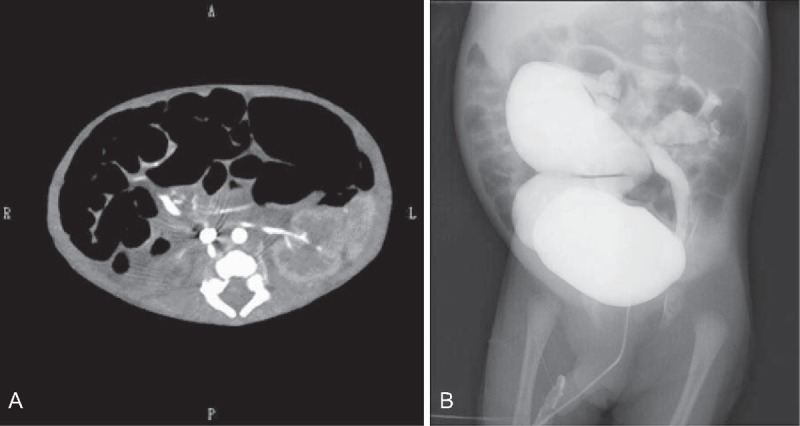
(A) CT image acquired at the level of the bilateral kidney. The right kidney of the patient was small compared with the left kidney. (B) VCUG revealing the VUR and dilation of the right renal pelvis and ureter. CT = computed tomography, VCUG = voiding cystourethrography, VUR = vesicoureteral reflux.

The right kidney of this patient was very small as detected by CT (Fig. 1A, Supplemental Fig. 1). The major axis of the right kidney was 17.7 mm, and that of the left kidney was 53.2 mm at 10 weeks old. The parenchyma of the right kidney was thin.

We obtained a DMSA scintigram (99mTc-DMSA) on the patient at the age of 6 weeks and found that the split renal function was 3:97 (right:left). Therefore, we diagnosed the right kidney as nonfunctioning. These findings demonstrated the indication for a right nephroureterectomy.

The patient also had severe VUR as detected by voiding cystourethrography (Fig. 1B). The voiding cystourethrography revealed 5th degree VUR in the right ureter and 4th degree VUR in the left ureter. The dilation of the right ureter was prominent.

We did not detect urethral lesions, including in the posterior urethral valve. We also did not detect bladder deformity when the bladder was full. The patient was able to urinate without residual urine. However, the refluxed urine immediately went down into the bladder and appeared as pseudo-residual urine.

Additionally, he had cryptorchidism and hyperdactylia.

Giemsa banding was done using the peripheral blood lymphocytes drawn from the infant's vein. 2 mL blood was drawn from the patient's vein. The lymphocytes were cultured for 72 hours with phytohemagglutinin for stimulation, and stopped the culture using Cocemid.

The patient had no siblings and his parents had no similar developmental abnormalities in genitourinary tract.

**Figure 2:**
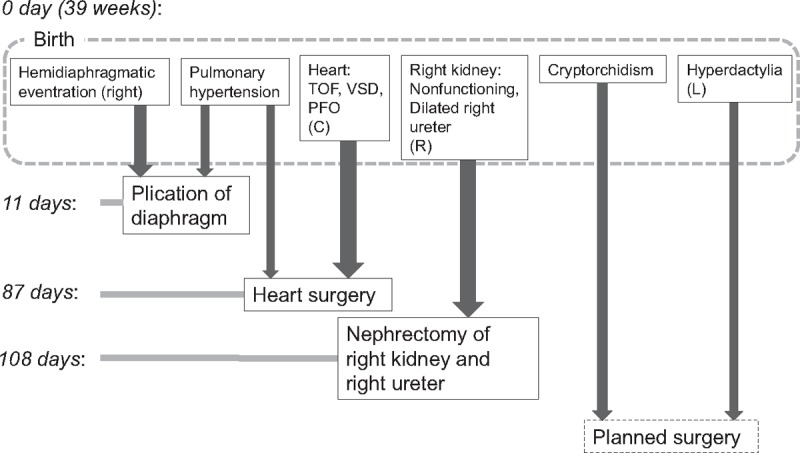
A flowchart illustrating the order of events related to the care of this patient.

The patient's treatments and the order in which they were applied were planned as follows based on discussions among the doctors; 1st, plication of diaphragm; 2nd, surgery for the congenital heart disease; 3rd, surgery for the renal and ureteral abnormalities; 4th, the surgery for the cryptorchidism; and 5th, plastic surgery for the hyperdactylia (Fig. 2).

First, the plication of the diaphragm via a thoracoscope was performed 11 days after birth.

The doctors discussed which surgery should be performed first, the heart surgery by the pediatric cardiac surgeons or the renal and vesicoureteral surgery by the pediatric urologists. The doctors prioritized the heart function, and the pediatric cardiac surgeons performed the heart surgery prior to the renal and vesicoureteral surgery.

Eighty-seven days after birth, the heart surgery was performed based on the doctors’ discussion. We predicted that the possibility of the spontaneous loss of the VUR in this patient was very low. Sustained high-grade VUR increases the risk of urinary tract infections.

Because the patient had a heart malformation that might have caused life-threatening infectious endocarditis or sepsis following a urinary tract infection, we decided to perform a nephroureterectomy on the patient.

Initially, we planned to perform the renal and vesicoureteral surgery after total recovery from the heart surgery. However, the patient exhibited a feverish urinary tract infection 8 days after the heart surgery. To prevent the infection from advancing in severity, we performed the nephrostomy of the right kidney and waited for the recovery from inflammation.

Long-term renal catheterization might increase the risk of urinary tract infection and its progression into infectious endocarditis. Therefore, the pediatric urologists and other doctors decided that the origin of the urinary tract infection should be removed as soon as possible. Therefore, we performed the renal and vesicoureteral surgery 21 days after the heart surgery and waited for the recovery from the feverish urinary tract infection.

We performed the nephroureterectomy of right kidney and right ureter and a procedure to prevent the backflow in the left ureter at 108 days after birth.

We performed open abdominal surgery. We opened the right flank and removed the right kidney and upper right ureter. Next, we opened the hypogastrium and removed the lower right ureter and performed the surgery to prevent the VUR on the left side. The surgical specimens of the right kidney and ureter were subjected to histopathological examinations.

The creatinine score was 0.24 mg/dL at 13 weeks after the right nephroureterectomy, and this did not represent an increase relative to the preoperative score.

The prognoses for the respiratory, cardiac, and renal functions of this patient were good. The pediatric cardiac surgeons are planning to perform the subsequent cardiac surgery when the patient's weight increases.

Informed consent was given for this study.

### 2.2 Histopathological findings

**Figure 3:**
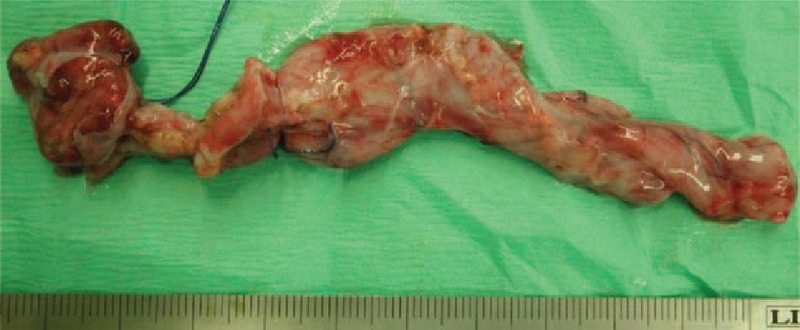
Macroscopic view of the surgical specimen. The right kidney was small and atrophic (left side of the figure). The right ureter was dilated due to vesicoureteral reflux (VUR, right side of the figure).

The right kidney was 30 × 20 × 10 mm and very small and atrophic (Fig. 3).

The renal pelvis was dilated, and the wall of the renal pelvis was thick (Fig. 3).

The renal parenchyma exhibited partial fibrosis with lymphocyte infiltration. In the fibrotic part, concentric circular fibromuscular coats had formed around the lumina of the collecting tubules.

**Figure 4:**
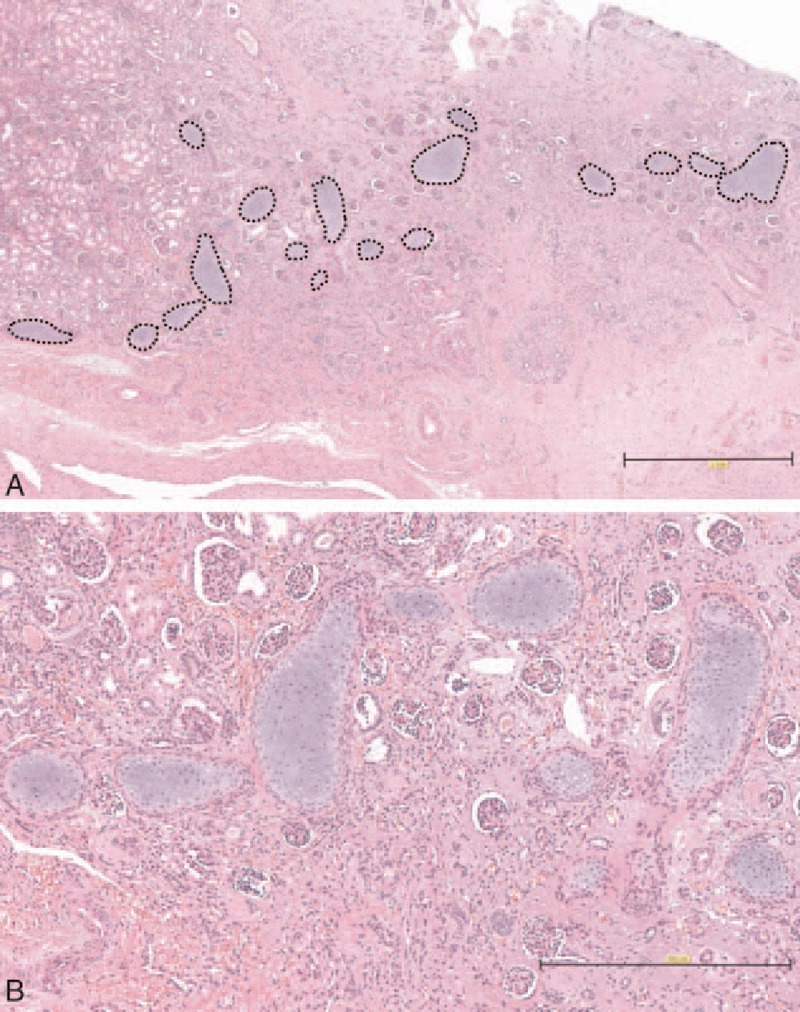
(A) The right nonfunctioning kidney included a prominent number of cartilaginous metaplasia lesions surrounded with dashed lines. (B) Magnified picture of the cartilaginous metaplasia lesions. The cartilaginous metaplasia lesions consisted of mature cartilaginous components.

In the renal parenchyma, we found cartilaginous islands surrounded by fibroblasts. The densities of the ectopic cartilaginous lesions in the nonfunctioning kidney of this case were extraordinarily high compared with typical renal dysplasia cases (Fig. 4A). The ectopic cartilaginous lesions were mature and included no immature components (Fig. 4B).

We also detected some small calcifications in the right kidney by histological examination.

We did not detect immature components suggestive of intralobar nephrogenic rests.

Although the right kidney was small and nonfunctioning, it included mature glomeruli and renal tubules on microscopic examination.

The right ureter was expanded, but the ureter mucosa did not include atypical cells.

## 3 Discussion

The existence of cartilaginous metaplasia is not rare in renal dysplasia.^[1,7]^ However, the number and density of the mature cartilaginous islands in this case of renal dysplasia were prominently high compared with typical cartilaginous metaplasia associated with renal dysplasia cases (Fig. 4A).

The 1st possibility is that this case occurred to have very prominent cartilaginous metaplasia due to renal dysplasia. An enlarged renal pelvis suggests the possibility of an ureteropelvic junction obstruction, which, when severe, can be associated with the loss of renal function.

The 2nd possibility is that the prominent cartilaginous metaplasia was associated with multiple developmental abnormalities, that is, VACTERL association, in this patient.

Because the patient has not undergone a detailed genetic investigation due to the refusal of the parents, we were unable to detect any precise genomic cause of this patient's constellation of symptoms. The patient did not have obvious structural or number abnormality of chromosomes by the Giemsa banding examination.

VACTERL association is defined as the combination of at least 3 of the following congenital anomalies: vertebral defects (V), anorectal malformations (A), cardiac defects (C), tracheoesophageal fistula with or without esophageal atresia (TE), renal malformations (R), and limb defects (L).^[5,6,8,9]^ The patient had cardiac defects (C), renal malformations (R), and limb defects (L). This combination of developmental anomalies satisfied the diagnostic criteria for VACTERL association.

De novo microduplications at 1q41, 2q37.3, and 8q24.3 have been discovered in patients with VACTERL association, and the *GPR35* gene is a candidate gene for VACTERL association.^[5]^

Single-gene disorders resembling the VACTERL association have been reported and include the following: the *N-Myc* gene in Feingold syndrome,^[10,11]^ the *CHD7* gene in CHARGE syndrome,^[12]^ the *FANC* family of genes in Fanconi anemia,^[13,14]^ the *SALL1* gene in Townes–Brocks syndrome,^[15]^ and the *GLI3* gene in Pallister–Hall syndrome.^[16]^

However, the identification of the disease-causing gene in VACTERL association has not been accomplished.^[5]^

This case might represent a novel type of VACTERL association that manifests as a combination of renal dysplasia with prominent cartilaginous metaplasia, tetralogy of Fallot and VSD of the heart, hemidiaphragmatic eventration, and hyperdactylia. The patient may have novel genomic abnormalities that have not been previously discovered.

Some cases of renal dysplasia are associated with gene abnormalities, such as abnormalities of the *paired box gene 2* (*PAX2*), *paired box gene 8* (*PAX8*), *Wilms tumor 1* (*WT1*), and *B-cell lymphoma 2* (*BCL2*) genes.^[17,18]^ The patient might have mutations in these genes.

In addition, renal dysplasia-associated syndromes have been reported.

Meckel syndrome is characterized by renal cystic dysplasia, pulmonary hypoplasia, hepatic developmental defects, encephalocele, and polydactyly. The ciliary gene *RPGRIP1L* is mutated in cerebello-oculo-renal syndrome (Joubert syndrome type B) and Meckel syndrome.^[19]^

Renal dysplasia-associated syndromes include VACTERL association in addition to Renal-coloboma syndrome, Prune belly syndrome, dysplasia renal coloboma syndrome, Herlyn–Werner–Wunderlich syndrome, branchio-oto-renal dysplasia, renal-hepatic-pancreatic dysplasia, and multiple endocrine neoplasia type 2A.^[20–27]^

The patient might have the known and possible genetic causes (chromosomal abnormalities and single gene mutations) for renal dysplasia, renal dysplasia-associated syndromes, as described above.

During development, the heart is derived from the mesoderm. The kidneys are also derived from the mesoderm during development. The thoracic diaphragm is derived from the somatic mesoderm. The patient might have some genomic abnormalities that affected the control of the development of organs from the mesoderm.

This case provides new insight into multiple developmental abnormalities through prominent cartilaginous lesions in the kidney.

## Acknowledgements

The authors thank the members of the Departments of Pathology and Pediatric Urology of Jichi Medical University for their support and help.

## Supplementary Material

Supplemental Digital Content
