# Supplementary figures and images for: Renal dysplasia characterized by prominent cartilaginous metaplasia lesions in VACTERL association: A case report
Source: Medicine (Baltimore). 2017 Apr 14;96(15):e6499. doi: 10.1097/MD.0000000000006499 (PMC5403075; doi:10.1097/MD.0000000000006499)

## Slide 1
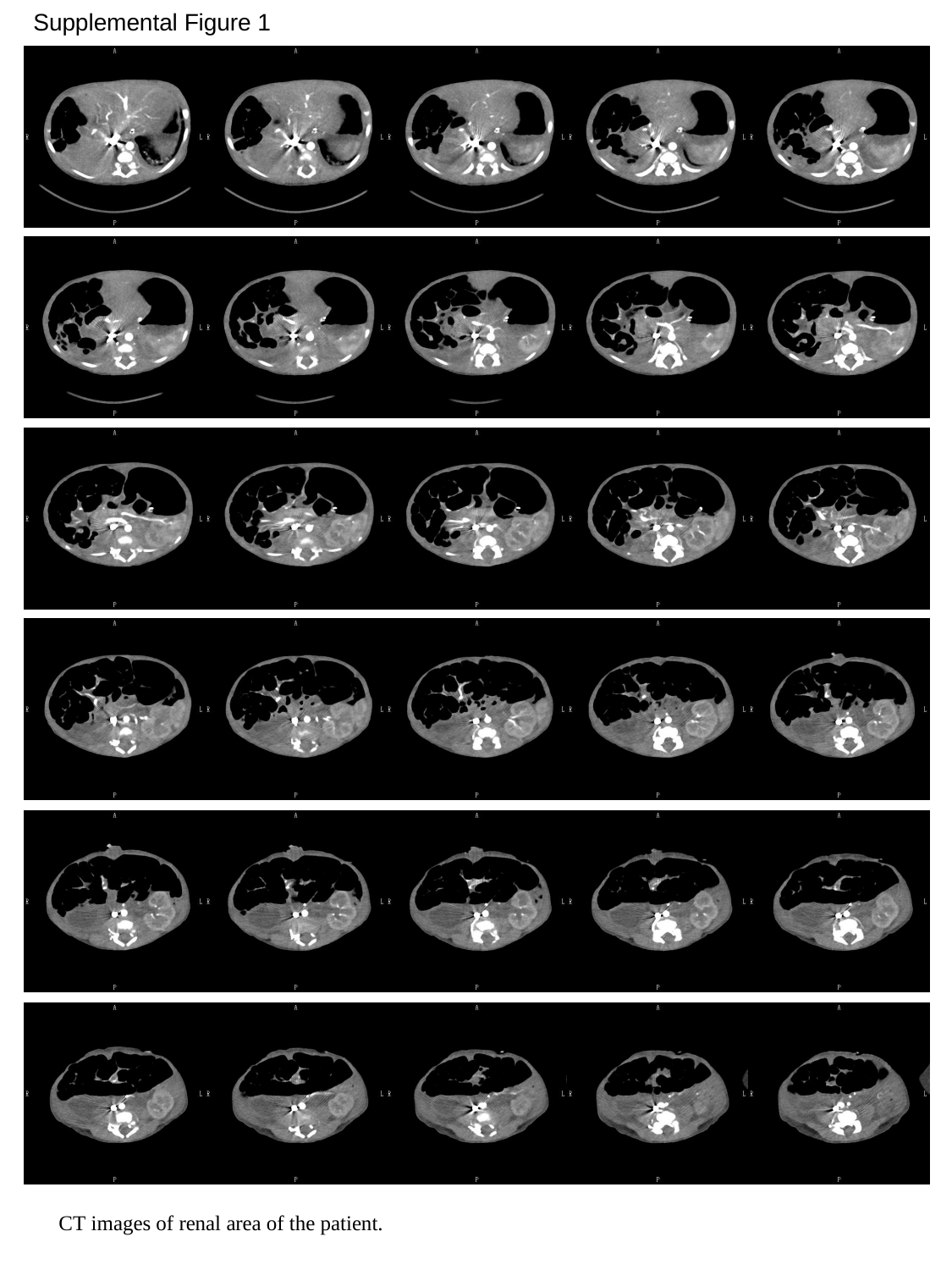

Supplemental Figure 1
#
CT images of renal area of the patient.

Supplement: Supplemental Digital Content [file medi-96-e6499-s001.pptx]
